# The Molecular Mechanisms and Therapeutic Potential of Cranberry, D-Mannose, and Flavonoids against Infectious Diseases: The Example of Urinary Tract Infections

**DOI:** 10.3390/antibiotics13070593

**Published:** 2024-06-26

**Authors:** Petros Ioannou, Stella Baliou

**Affiliations:** School of Medicine, University of Crete, 71003 Heraklion, Greece

**Keywords:** cranberry, D-mannose, infectious diseases, polyphenols, flavonoids

## Abstract

The treatment of infectious diseases typically includes the administration of anti-infectives; however, the increasing rates of antimicrobial resistance (AMR) have led to attempts to develop other modalities, such as antimicrobial peptides, nanotechnology, bacteriophages, and natural products. Natural products offer a viable alternative due to their potential affordability, ease of access, and diverse biological activities. Flavonoids, a class of natural polyphenols, demonstrate broad anti-infective properties against viruses, bacteria, fungi, and parasites. Their mechanisms of action include disruption of microbial membranes, inhibition of nucleic acid synthesis, and interference with bacterial enzymes. This review explores the potential of natural compounds, such as flavonoids, as an alternative therapeutic approach to combat infectious diseases. Moreover, it discusses some commonly used natural products, such as cranberry and D-mannose, to manage urinary tract infections (UTIs). Cranberry products and D-mannose both, yet differently, inhibit the adhesion of uropathogenic bacteria to the urothelium, thus reducing the likelihood of UTI occurrence. Some studies, with methodological limitations and small patient samples, provide some encouraging results suggesting the use of these substances in the prevention of recurrent UTIs. While further research is needed to determine optimal dosages, bioavailability, and potential side effects, natural compounds hold promise as a complementary or alternative therapeutic strategy in the fight against infectious diseases.

## 1. Introduction

Infectious diseases can be caused by bacteria, viruses, fungi, parasites, or other infectious factors, such as prions [1]. Their treatment classically includes the administration of anti-infectives, such as antibiotics, antivirals, antifungals, or antiparasitic drugs [1]. However, antimicrobial resistance (AMR) is emerging worldwide. It can be a considerable cause of morbidity and mortality in patients suffering from infections from pathogens with significant AMR, such as multi-drug-resistant (MDR) pathogens, extensively-resistant pathogens (XDR), and pan-drug-resistant (PDR) pathogens [2,3].

The development of AMR has led to the need to develop novel methods for treating infections that may not involve anti-infectives. These anti-infective-sparing techniques can involve several methods, such as antimicrobial peptides, bacteriophages, nanotechnology, or other methods [4,5,6]. In most cases, these methods are at the stage of clinical studies and have not yet been approved. Moreover, these products may be technologically demanding, requiring advanced techniques for design, production, and effective and safe use.

On the other hand, substances from natural products and simple chemical compounds could have anti-infective activity and, in some cases, be used to manage simple infections, such as urinary tract infections [7]. The production of such substances could be less laborious while they could be equally safe as more complex and technologically demanding substances [8]. Such substances, such as cranberry products or D-mannose, have been suggested for the management of recurrent urinary tract infections (UTIs) in the past; however, there is no consensus for supporting their broad use currently [9].

The present study aimed to review the theoretical role of natural compounds, and more specifically, of flavonoids in treating infectious diseases and also to evaluate the role of cranberries and D-mannose in treating UTIs.

## 2. The Need for and Examples of Alternative Treatments in the Treatment of Infectious Diseases

The increasing rates of AMR among bacterial pathogens are worrisome, and the trends suggest that AMR will be a considerable cause of mortality in the following decades, leaving few anti-infectives available as viable options for treating infections [10,11]. It is estimated that in 2050, if no action is taken, AMR could even be a primary cause of death [12]. The Global Burden of bacterial antimicrobial resistance study that was published in 2022 showed that an estimated 4.95 million deaths in 2019 were associated with bacterial AMR [3]. In terms of geographical distribution, the all-death rate attributable to AMR was estimated to be higher in western sub-Saharan Africa and lowest in Australia [3]. Several pathogens may have significant AMR that is of particular concern from a public health perspective. For example, MDR tuberculosis (infection by *Mycobacterium tuberculosis* with resistance to rifampicin and isoniazid), an infectious disease with high transmissibility, is common in Asia, while one of the most common resistant Gram-positive bacteria, methicillin-resistant *Staphylococcus aureus* (MRSA), is increasingly prevalent in North and South America, South Europe, Asia, and some countries in Africa, with MRSA being associated with higher mortality rates compared to methicillin-susceptible *S. aureus* (MSSA) [3,13,14]. Moreover, *Escherichia coli*, the most common cause of UTIs and one of the most commonly identified Gram-negative bacteria in microbiology laboratories, has increased rates of resistance in the last decades, with very high rates of ampicillin resistance, extended-spectrum beta lactamase production, and even increasing rates of carbapenem resistance among these and other Enterobacteriaceae species [15,16,17,18]. To that end, the prevalence of AMR *E. coli* has almost doubled from 2001 to 2010 and keeps rising [19]. Areas like South Asia, Central America, and South Europe have increased rates of *E. coli* strains with resistance to third-generation cephalosporins [3].

The rise of AMR can be associated with huge surcharges, leading to an increased cost of many thousands of US dollars per patient episode, thus causing a substantial burden to the healthcare system and the global economy [20]. Moreover, the rise of AMR can lead to increased consumption of anti-infectives in medicine and other fields, such as animal husbandry, aquaculture, food preservation, and veterinary medicine among others [21]. Indeed, this magnitude of uses of anti-infectives can lead to the selection of resistant species of microorganisms [21]. Moreover, antimicrobial resistance can also spread through mobile genetic elements, as in the case of plasmid transfer through conjugation, transduction with phages, or transformation through uptake of free DNA [22]. Probiotics, on the other hand, are live microorganisms that could protect against infectious diseases, most commonly through a reduction in the likelihood of development of infection through various mechanisms, such as competitive inhibition, immunomodulation, production of mediators such as defensins or other anti-infective compounds, and inhibition of biofilm formation [22]. Even though their role is ambiguous for some conditions, such as *Clostridioides difficile* infection, there are studies suggesting their potential benefit in other instances, even though more research is needed [23]. For example, a recent systematic review showed that probiotic and symbiotic supplements could reduce the likelihood of ventilator-associated pneumonia and sepsis in critically ill patients, reduce the duration of intensive care unit (ICU) and hospital stay, and lower mortality in the ICU [24].

Thus, using methods other than anti-infectives could be associated with benefits such as the lack of development of resistance, the reduced likelihood of adverse reactions, and the possibility of drug delivery precisely to the site of infection. For example, organisms naturally produce antimicrobial peptides to protect them against pathogens, and these peptides could be used to treat infections. Some of them, such as daptomycin and colistin have already been approved for human use, while others are currently used in fields other than medicine, such as food preservatives [5]. Bacteriophages are organisms that exist naturally and target bacteria. This interaction could be exploited to allow their use as anti-infective factors in treating infectious diseases. This could be beneficial since they could have minimal effects on the host’s microbiome and have the theoretical advantage of not causing adverse effects. However, although they have several non-medical applications, they have not been approved for human use. At the same time, their production could be technically demanding since they have high selectivity for their target bacteria [4]. Nanotechnology has been mainly exploited for diagnostic purposes in infectious diseases; however, their use in treating infections is highly demanding from a technical perspective, even though they could provide nanobiotics that could deliver the antibiotic substance precisely at the site of infection [6].

## 3. Literature Search Methodology

To review the role of natural compounds, and, more specifically, of flavonoids in the management of infectious diseases, and also evaluate the role of cranberry products and D-mannose in the treatment of UTIs, a search of the PubMed/Medline database up until 30 April 2024 for eligible articles was conducted. The search term ‘(flavonoids OR D-mannose OR cranberry) AND infection’ was used. Relevant reviews and original studies providing data on the topic were retrieved, evaluated, and added to the synthesis in a liberal way. The two investigators (PI and SB) screened the studies and extracted and synthesized the evidence. The references of the included articles were searched to identify other relevant articles.

## 4. The Potential of Flavonoids in the Management of Infectious Diseases and Their Underlying Molecular Mechanisms

In many low- and middle-income countries, growing health expenditure is associated with a growing inaccessibility to treatment [25]. On the one hand, increasing AMR and the growing cost associated with it could lead to difficulties in providing treatments [3,20]. On the other hand, natural remedies may be inexpensive and more readily available [26]. In the case of infectious diseases, natural products seem promising because they reduce the burden of pathogens and restore the balance of healthy microbiota. Moreover, they can also suppress or eradicate biofilms [26,27].

Flavonoids are natural polyphenols synthesized by plants, and they have many distinct categories, including isoflavones (daidzein and genistein), flavones (luteolin and apigenin), flavonols (galangin, quercetin, and kaempferol), flavanones (naringerin and hesperetin), flavanonols (taxifolin), and flavan-3-ols (catechin and epicatechin), which are precursors of tannins—catechins [28]. Throughout the plant kingdom, flavonoids are physiologically active phytochemicals used in various herbal remedies, and they constitute the most prevalent and widely distributed phytochemicals in herbs [29]. In foods, flavonoids can be detected in tea, wine, grains, fruits, vegetables, and bark [30]. Accordingly, flavones can be detected in chamomile, red peppers, celery, parsley, mint, and Ginkgo biloba, exerting anticancer, antioxidant, and anti-inflammatory properties. Flavanone-rich foods include grapes, grapefruit, lemons, limes, and oranges [31]. Due to their antioxidant properties, flavones and flavonols are crucial for shielding plants from UV rays [32,33]. Of particular interest, the main flavone in food is luteolin. Cereals and herbs contain luteolin, while vegetables like broccoli and carrots have glycosylated luteolin [34,35]. Luteolin has tumor-suppressive properties, hindering oxidative stress and inflammation and modulating gut microbiota [36,37]. In addition, isoflavonoids from lentils, peas, beans, tofu, and soybeans have been demonstrated to ameliorate vascular complications. Anthocyanins are another category of flavonoids involved in food types (eggplant, red cabbage, blood oranges, cherries, black plums, elderberries, blackberries, strawberries, cranberries, red grapes, and red wine). In this context, the anthocyanins have also proved effective in sustaining cardiovascular health. Accordingly, flavonoid supplements like silymarin, green tea extracts, rutin, curcumin, and quercetin display similar properties [31]. Quercetin, whose chemical name is 2-(3,4-dihydroxyphenyl)-3,5,7-trihydroxychromen-4-one, is a flavonoid studied extensively. Many fruits (apples, berries, etc.) and vegetables (lettuce, broccoli, tomato, etc.) provide daily sources of quercetin in its glycoside type. From a molecular perspective, quercetin has an anticoagulant, antiproliferative, anti-infective, and antioxidant nature that is advantageous for health and longevity [38]. Quercetin has been shown to hinder growth inhibition of the following bacterial species, including *S. sobrinus*, *L. acidophilus*, *S. sanguis*, *A. actinomycetemocomitans*, and *P. intermedia* [39]. Several examples substantiate the action of quercetin to modulate the action of F-type ATPase across the membrane [39].

Flavonoids have anticancer, anti-inflammatory, antioxidant, antibacterial, antiviral, anticancer, antidiabetic, and wound-healing properties [34,35,36]. Notably, researchers have emphasized the anti-inflammatory effect of flavonoids in managing diseases. Indeed, they seem to reduce inflammation in leukemia, sepsis, asthma, sclerosis, atherosclerosis, psoriasis, allergic rhinitis, ileitis/colitis, and rheumatoid arthritis, attenuating their clinical signs [40,41]. In particular, flavonoids seem to interfere with the production of proinflammatory cytokines, chemokines, plasma proteases, prostaglandins, leukotrienes, interleukins, and nitric oxide [42,43]. For example, Chen et al. have supported that flavonoids suppressed the synthesis of prostaglandin E_2_ (PGE_2_), nitric oxide (NO), and interleukin-6 (IL-6) in LPS-induced macrophages [42,43]. In another case, it has been documented that quercetin prevents nuclear factor-kappa B (NF-kB) transcriptional translocation in intestinal epithelial cells, thus, hindering acute inflammation [42,43].

A growing body of research has focused on elucidating the antiviral effects of flavonoids against multiple viruses. For example, the suppressive action of flavonoids against the human immunodeficiency virus (HIV) is a significant field of research. For example, Li et al. have also shown that the flavone O-glycoside inhibits HIV-1 entrance into cells expressing CD4 and chemokine co-receptors [44], and it antagonizes HIV-1 reverse transcriptase [44]. In addition to the above, other studies have shown that numerous catechins, including robustaflavone, hinokiflavone, and baicalein, can inhibit HIV-1 reverse transcriptase [45]. Moreover, dietary flavonoids are considered promising for the treatment of coronavirus disease 2019 (COVID-19), and they have been shown to have direct antiviral properties against influenza, Ebola, severe acute respiratory syndrome (SARS), and Middle East respiratory syndrome (MERS) [46,47,48].

In addition, the antiparasitic effects of flavonoids have also been examined. For example, flavonoids have been proven very promising in the defense against the parasite *Entaemoba histolytica* trophozoites [49]. While further studies are needed to figure out the precise molecular targets, bioavailability, and administration method of flavonoids, they offer a very exciting and practical approach that should be taken into consideration as protection against *E. histolytica* [49].

### 4.1. The Antibacterial Impact of Flavonoids and Their Underlying Mechanism

The microbicidal activity of flavonoids has received considerable attention. Several examples have substantiated the beneficial effect of flavonoids in managing bacterial infections. Initially, a methanolic extract of the leaves of *B. purpurascens* proved effective in combating some microbial pathogens. This medicinal herb contains a variety of flavonoids, including bergenin, catechin, naringenin, and myricetin. This methanolic extract also showed strong antibacterial effects against *S. aureus* and *Streptococcus* species [31]. In another case, 3,3,4-trihydroxyflavone found in the methanolic fraction of *Justicia wynaadensis* extract had antibacterial efficacy against opportunistic and multi-resistant pathogens. *Enterococcus faecalis*, *Klebsiella pneumoniae*, *Enterobacter aerogenes*, *Escherichia coli*, and *Pseudomonas aeruginosa* are some bacteria which flavones efficiently killed in diabetic wounds [50]. For example, a recent systematic review highlighted the effect of flavonoids on the development of upper respiratory tract infection (URTI). In particular, flavonoid supplementation decreased the onset of URTI by 33% without causing any apparent side effects [51].

**Figure 1:**
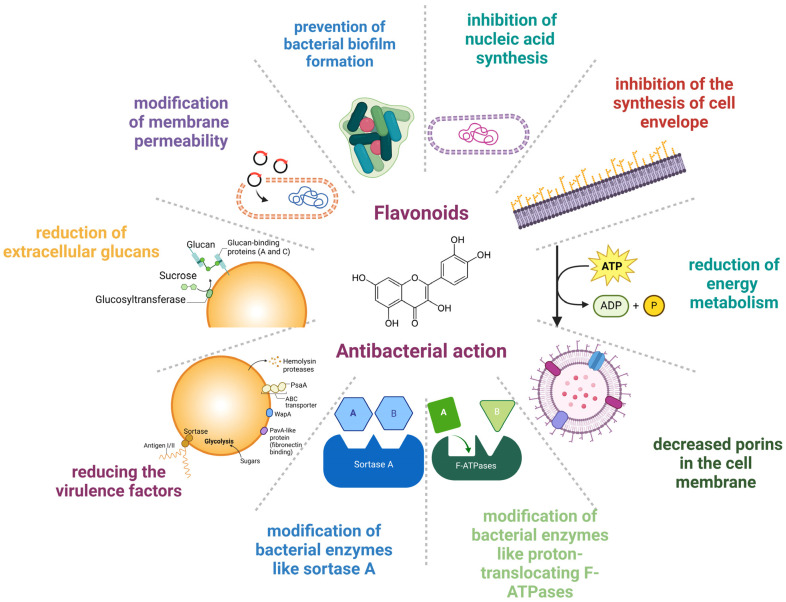
Mechanisms underlying the anti-infective effect of flavonoids. Created with BioRender.com (license date: 29 May 2024).

From a molecular perspective, the mechanisms underlying the antibacterial activity of flavonoids are the following: modification of membrane permeability, decreased porins in the cell membrane, prevention of bacterial biofilm formation, inhibition of nucleic acid synthesis, inhibition of the synthesis of the cell envelope, reduction in energy metabolism, hindering virulence factors, prevention of extracellular glycans, and changes in bacterial enzymes such as sortase A and proton-translocating F-ATPases [27,28,52]. Other underlying mechanisms that account for the protective effects of flavonoids rely on the modification of bacterial enzymes like proton-translocating F-ATPases and sortase A (SrtA) and on the synthesis of extracellular glucans by glucosyltransferase (GTF), thus reducing the virulence factors [53]. Figure 1 shows the mechanisms of the anti-infective effects of flavonoids.

In more detail, flavonoids have been used in several ways to reduce bacterial growth. For example, flavonoids can interfere with membrane lipid bilayers in two ways, thereby contributing to membrane rupture [54]. The first one involves the more non-polar molecules splitting apart in the hydrophobic membrane’s interior, and the second one involves hydrogen bonds forming between lipids’ polar head groups and the more hydrophilic flavonoids at the membrane. Several examples of flavonoids have substantiated their capacity to cause membrane rupture. Initially, catechins have been illustrated to cause membrane disruption by hindering the action of membrane enzymes [55]. Other polyphenols, such as the salidroside, rutin (quercetin-3-O-rhamnoglucoside), and quercetin, have been shown to cause membrane damage [56]. Epicatechin (EC), epigallocatechin gallate (EGCG), and a flavonol quercetin are some flavonoid cases with reported membrane damage [57]. Epigallocatechin gallate (EGCG) is the major catechin involved in green tea and is also present in legumes and fruits [58,59]. Epicatechin (EC) can be found in grapes, blackberries, cherries, apples, raspberries, pears, broad beans, cocoa, and tea leaves [60]. The bactericidal properties of EGCG and EC have been attributed to their membrane-disrupting nature [61]. Apart from the membrane disruption, the microbicidal effect of flavonoids can also be attributed to their pro-oxidant properties. According to Fathima and Rao, catechins have been shown to eliminate bacterial growth due to an oxidative burst [57]. In particular, catechins caused the excessive formation of reactive oxygen species (ROS), which in turn modified membrane permeability and induced membrane rupture [57].

Thirdly, the flavonoids’ antibacterial effect has been explained by their capacity to prevent biofilm formation. For example, synthetic lipophilic 3-arylideneflavonones (replaced with different phenolic compounds at the C-3 position of the C Ring) are highly effective against *S. aureus*, *Staphylococcus epidermidis*, and *Enterococcus faecalis* due to their capacity to inhibit biofilm formation [62].

Moreover, flavonoids’ ability to interfere with nucleic acid synthesis seems to constitute another mechanism underlying their protective action against bacterial pathogens. To support this, some examples of relevant flavonoids have been reported to be myricetin, robinet, and (−)-epigallocatechin, which have been considered to account for the prevention of DNA synthesis. In more detail, the negative impact of flavonoids on DNA and RNA synthesis can be explained by the possibility that the B ring of the flavonoids is involved in intercalation or hydrogen bonding with the stacking of nucleic acid bases [63].

In addition to the above, the antibacterial impact of flavonoids can be the result of their ability to hinder the activity of DNA enzymes. Indeed, polyphenols like quercetin, apigenin, and 3,6,7,3′,4′-pentahydroxyflavone have been proven to hinder bacterial growth of *S. epidermidis*, *S. aureus*, *E. coli*, *S. typhimurium*, and *Stenotrophomonas maltophilia* by limiting the action of DNA gyrase [64].

In addition, flavonoids have been reported to reduce energy metabolism. Some flavonoids have shown suppressive action against the bacterial growth of *S. aureus* and *Micrococcus luteus* but not against *E. coli*. In particular, licochalcones were revealed to hinder oxygen consumption in *M. luteus* and *S. aureus* but not in *E. coli*. Interestingly, licochalcones seemed to interfere with energy metabolism similar to respiratory-inhibiting antibiotics [65]. In this direction, it has been highlighted that flavonoids can downregulate the electron transport chain, hindering ATP synthesis. Various polyphenols have been demonstrated to bind to ATP synthase’s unique polyphenol binding site. Since the polyphenol binding pocket is located at the interface between the F1 sector’s a, b, and c subunits of ATPase, polyphenols seem to inhibit the action of ATP synthase. In particular, the speculated route of flavonoid inhibitory properties is mediated through binding to ATP synthase’s polyphenol binding pocket and preventing the rotation of the ATP synthase c-subunit [66]. Recently, it came to light that flavonoids may interfere with *E. coli* F1FO ATPase [67]. Consistent with the above, Plaper and colleagues have also shown that quercetin can suppress the ATPase activity of *E. coli* DNA gyrase by binding to its GyrB component [68], taking into consideration that quercetin binds to the B subunit of gyrase. Indeed, quercetin blocks the ATP binding pocket by generating hydrogen bonds via 5, 7, and 30 -OH groups to the amino acid residues of DNA gyrase [68]. Accordingly, some flavonoids have been reported to hinder the ATP binding pocket of D-alanine-D-alanine ligase [69].

### 4.2. The Synergistic Effect of Flavonoids with Antibiotics in the Fight against Antimicrobial Resistance

Flavonoids have received considerable attention recently as the main antibiotic substitute since AMR has rendered increasing numbers of microbial illnesses resistant to treatment [26]. Specific flavonoids can enhance the therapeutic value of the antibiotics’ resistance. [27]. Due to the rise of drug-resistant bacteria, novel anti-infective agents have been identified. In this regard, flavonoids are considered great options to combat drug-resistant microbes, given the broad spectrum of their biological activities. Unlike Gram-positive bacteria, Gram-negative bacteria are resistant to a wide spectrum of antibiotics, owing to the limited permeability of their cell membrane. The key mechanism linked to their resistance is MexAB-OprM and AcrAB-TolC efflux pumps, as well as poor porin expression [70]. The increased secretion of antibiotics through efflux pumps is prevalent in MDR bacteria [5]. While flavonoids present in nature have been extensively studied, semi-synthetic or synthetic flavonoids have also been appreciated as promising therapeutics in preventing or even eliminating bacteria at concentrations lower than 1 μg/mL. Synthetic flavonoids feature a substituted pattern that frequently contains hydroxy groups, halogens, or other heteroatomic rings like cations of 1,3-dithiolium, piperidine, or pyridine [71].

Flavonoids in combination with anti-infectives can be used to combat AMR. In this direction, the flavonoid dihydromyricetin (DHM) and the two triterpenoids ursolic acid (UA) and oleanolic acid (OA) were examined for their ability to counteract bacterial growth [72]. Without adversely affecting eukaryotic cells, all of the substances displayed antibacterial capacity against Gram-negative bacteria (*Proteus hauseri*, *E. coli*, and *Campylobacter jejuni*) and Gram-positive bacteria (*S. aureus*, *S. epidermidis*, and *Listeria monocytogenes*). Related to Gram-positive types of bacteria, the triterpenoids demonstrated the most potent antibacterial activity. The same study highlighted that flavonoid dihydromyricetin (DHM) can exert therapeutic benefit against *Staphylococcus* strains when combined with two triterpenoids, ursolic acid (UA) and oleanolic acid (OA) [72]. As a result, DHM’s antibacterial activity was strengthened when combined with triterpenoids [72].

Combining Quercetin-3-arabinofuranoside with Myricetin with Procyanidin A2 exerts bactericidal action against *S. mutans* and *S. anginosus*, by hindering the action of glucosyltransferases and F-ATPases [73].

Another combination of the following substances, Luteolin, Morin, Naringin, Quercetin, and Rutin, has been proven helpful in combating *A. naeslundii*, *A. viscosus*, *A. actinomycecomitans*, *E. faecalis*, and *L. casei* [74].

Flavonoids seem beneficial in mitigating candidiasis caused by *Candida albicans*, a fungus frequently inhabiting the oral mucosa. Flavonoids combat antimicrobial-resistant candidiasis by escaping resistance to fluconazole [75]. Apart from fluconazole, other therapeutic options are polyenes, azoles, and echinocandin. Many commercial pharmaceuticals used to treat candidiasis are in the azole class; therefore, the activity of flavonoids will be compared to that of recently synthesized azole compounds and to azole medications currently available as approved medications [76]. In particular, combining certain flavonoids with antifungal azole drugs can exhibit therapeutic benefits against antibiotic-resistant *Candida albicans* [77]. The flavonoids potentiate the therapeutic efficacy of azole drugs, modulating their bioavailability and metabolism, thus contributing to the attenuation of candidiasis [77]. Similarly, quercetin can act synergistically with fluconazole to exhibit antifungal action against *C. albicans* [78]. The mechanisms underlying the therapeutic potential of the aforementioned therapeutics are based on the induction of cell death and DNA damage [78].

From a molecular perspective, flavonoids have been shown to increase antibiotic efficacy by boosting endogenous ROS formation, which might compromise the organism’s ability to deal with oxidative stress triggered by the antibiotic. For example, antibiotics like quinolones, b-lactams, and aminoglycosides are known to account for hydroxyl radical generation via the Fenton reaction [79]. Accordingly, the flavonoids served as either hydroxyl radical quenchers or hydroxyl radical quenchers seem to reduce bacterial killing, implying that oxidative burst through hydroxyl radical formation can amplify antibiotic-induced cell death [79]. Table 1 shows some examples of studies that provide data on the activity of different flavonoids against commonly isolated microorganisms, with some of them also suggesting synergy with specific antibiotics.

### 4.3. Clinical Trials of Flavonoids in Infectious Diseases

Flavonoids have not only been evaluated in vitro. Several clinical studies evaluate their efficacy and safety in managing acute respiratory tract infections. For example, a recent systematic review evaluated 30 randomized controlled trials (RCTs) providing data for more than 5000 patients with respiratory tract infections [94]. The results from the meta-analysis of this systematic review suggested that flavonoids were safe and had adequate clinical efficacy in treating viral acute respiratory tract infections, including COVID-19, influenza, common cold, acute rhinosinusitis, acute non-streptococcal tonsillopharyngitis, acute bronchitis, pneumonia, and infections of the upper respiratory tract. Yet, as the authors of this systematic review also stated, their results should be read cautiously since there were few RCTs per type of acute respiratory tract infection. At the same time, many RCTs had a significant risk of bias and were of low quality. Thus, larger studies should be conducted to draw safe conclusions regarding flavonoids’ effect on these infections.

In another study, the effect of luteolin 4′-Neohesperidoside against four antibiotic-resistant microorganisms was evaluated [95]. More specifically, in vitro studies showed that luteolin 4′-Neohesperidoside was effective against MRSA, *K. pneumoniae*, and Shiga toxin-producing *E. coli* (STEC) serogroup O111. Further experiments in vivo revealed a significant decrease in STEC and *K. pneumoniae* colonization and shedding. Those experiments implied that 4′-Neohesperidoside could be used as an antibiotic-sparing agent in patients, even with infections of resistant pathogens, if clinical trials in humans prove it is safe and efficient.

### 4.4. Limitations and Bioavailability of Flavonoids

The use of flavonoids may have some limitations. Initially, the daily consumption of flavonoids by food sources hinders their bioavailability. In particular, the absorption of flavonoids has been reported to be limited. For example, a study including 97 people revealed that the plasma concentrations of total polyphenolic metabolites were 0–4 μmol/L following 50 mg of aglycone equivalents, and the urinary excretion was 0.3–43% of the ingested dose [96]. Another issue is the purification or bioengineering of flavonoids remains difficult. Last but not least, developing optimal delivery formulations of flavonoids is critical to exert their therapeutic effect. For example, in a review by Thilakarathna et al. addressing issues regarding the bioavailability of flavonoids, an attempt to understand the necessary steps towards improving the bioavailability of flavonoids is made [97]. To that end, improving intestinal absorption using mixtures or delivery vehicles, such as lipid-based systems, could increase absorption for relatively insoluble molecules [98]. Another possibility could be using nanodelivery vehicles that could also lead to increased absorption of insoluble molecules [99]. Additionally, changing the site of absorption inside the gastrointestinal system via enzymatic conversion of the flavonoid could also lead to better absorption [100]. Increasing the metabolic stability of flavonoids through methylation or other modifications could also reduce their elimination after absorption [101]. However, future studies on pharmacokinetics and pharmacodynamics of flavonoids of particular interest in infectious diseases would be needed to allow a better understanding of their absorption, delivery, and elimination, as well as allow for their potential use against infections in the future.

## 5. Natural Products for the Management of Urinary Tract Infections

Recurrent UTIs, more commonly acute uncomplicated cystitis, can be a cause of considerable discomfort in women, more commonly before menopause. The definition of recurrent UTIs includes at least two infections within six months or at least three within one year. Recurrent simple cystitis is relatively frequent in young, healthy women without immunosuppression or abnormality in their anatomy and physiology of the urinary tract. At least 25% of female college students with an episode of UTI had a recurrence within the six months following the first episode, and isolation of E. coli (the most common cause of UTIs) was associated with a higher rate of recurrence [102,103]. Several risk factors such as sexual intercourse, spermicide use, having a first UTI at a young age, having a mother with a history of recurrent UTIs, urologic factors such as urinary incontinence or post-voiding residual urine, and other biological and genetic factors increase the risk for UTIs [104,105,106,107,108,109,110,111,112,113].

Management of patients with recurrent UTIs includes excluding reversible causes that increase the likelihood of infection, such as anatomical obstruction of urine flow, appropriate education about hygiene and practices that could reduce the infection rate, and in case these measures fail, treatment of patients with an agent that could reduce the infection rates [114]. Agents to reduce the recurrence rate of UTIs include using anti-infectives, such as the combination of trimethoprim and sulfamethoxazole, or nitrofurantoin, or non-anti-infective options, like natural products, such as cranberry products, or D-mannose [22]. The use of antibiotics is widely endorsed in the literature and in guidelines; however, adverse events may occur. For example, Clostridioides difficile colitis resulting from changes in the gut microbiome can develop in patients treated with antibiotics [115]. On the other hand, unnecessary use of antibiotics can lead to an increased risk of colonization and infection by pathogens with AMR [116]. Thus, the appropriate selection of the therapeutic agent to be used in patients with UTIs, especially when its symptoms are localized in the lower urinary tract, could be of clinical benefit to the patient and minimize unnecessary potential harm.

### 5.1. Cranberry Products

Cranberry fruit, which is abundant in polyphenols, exhibits a strong biological defense against *Streptococcus mutans*. In particular, the effects of cranberry extracts containing flavonols (FLAV), anthocyanins (A), and proanthocyanidins (PAC) were examined regarding the virulence parameters related to the formation of *Streptococcus mutans* biofilms and acidogenicity [117]. According to this research, the components of cranberries that effectively impeded *S. mutans* bacterial growth were flavonols and proanthocyanidins [117].

**Figure 2:**
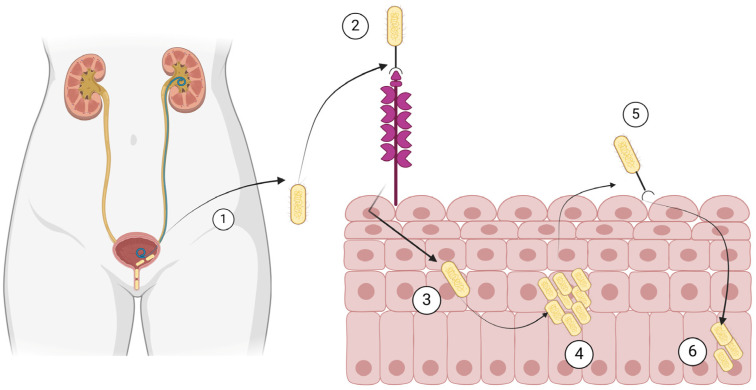
Pathogenesis of urinary tract infection by *Escherichia coli*. (1) *E. coli* ascends the urethra and (2) binds to mannose residues of uroplakins on the urothelium. (3) *E. coli* is then internalized, (4) it multiplies in the urothelial cells, (5) it recolonizes the bladder, and (6) it remains dormant in the urothelial cells. Created with BioRender.com (license date: 28 May 2024).

The most common use of cranberry products in infectious diseases involves their use in recurrent UTIs. Cranberry is a plant known as *Vaccinium macrocarpon*, *Vaccinium oxycoccos*, and *Vaccinium erythrocarpum*, in the family Ericaceae. They primarily consist of water and a mixture of fructose, organic acids, flavonoids, ascorbic acid, catechins, proanthocyanidins, anthocyanidins, and triterpenoids [22,118]. The factors considered more clinically relevant for preventing recurrent UTIs in women are anthocyanidins and proanthocyanidins, which are tannins (polyphenols) that function as natural plant defense against pathogens [22,119]. In the last centuries, before the discovery of antibiotics, cranberries had been used for UTIs and various other conditions, such as gastric and liver diseases, cancer, and scurvy [22,120,121]. The protection of cranberries against recurrent UTIs involves several mechanisms [22]. For example, cranberry juice is very acidic, and the associated acidification of urine has been proposed as a potential explanation of the protective effect of cranberry juice against recurrent UTIs [118,120]. A better understanding of the pathophysiology of UTIs and the role of some components of cranberries led to the identification of new potential mechanisms as well. For example, the adherence of bacterial cells on the urothelium, an important first step in the pathophysiology of a UTI, could be inhibited by cranberry products. More specifically, bacterial adhesins enable bacteria such as *E. coli* to bind to carbohydrate receptors on the surface of cells of the urothelium [22,121]. Such adhesins are inhibited by fructose and, maybe more importantly, proanthocyanidins, which are contained in cranberries [122]. Figure 2 shows the pathophysiology of UTIs that is relevant to the way the cranberry products work towards inhibition of UTI development.

Proanthocyanidins are polymers and oligomers of flavans, often linked by a single bond, even though, in cranberries, another bond (type A-linkage) can be found and is primarily implicated in the clinical effect of cranberries with respect to the reduction in recurrence of UTIs [123]. The specific association of A-linkage with this clinical effect was shown in human volunteers who consumed products containing different proanthocyanidins, such as grape juice, green tea, apple juice, and dark chocolate. The urine of the volunteers that had consumed cranberry juice had a higher ability to inhibit the adhesion of bacteria, as confirmed in vitro, and this would be attributed to the type A linkage of proanthocyanidins [123]. The exact mechanism that causes the reduced adhesion of bacteria to the urothelium in the presence of proanthocyanidins or other cranberry components is not yet completely defined, even though there are some proposed explanations [22]. For example, cranberry components can competitively inhibit the binding of *E. coli* on the urothelial cells by acting as analogs of those receptors [119]. Additionally, cranberry components could alter the properties of the cell surface of bacteria, thus affecting their ability to adhere to the urothelium. For example, bacterial cells of *E. coli* that have been exposed to cranberry compounds for long periods could present morphological changes, such as changing from rod shaped to spherical [124]. Other compounds of cranberries could also have similar properties. For example, ursolic acid (a pentacyclic triterpenoid) may have a synergistic or complementary role to proanthocyanidins towards the inhibition of bacterial adhesion through altering gene expression and inhibiting the formation of biofilms [125].

Cranberries can be administered in many forms, such as tablets, juice cocktails, or fresh berries. However, the concentration of proanthocyanidins differs between these different forms, and the precise content of the original preparation exists only in whole cranberry fruit [22,126]. It has been estimated that 240 mL of cranberry juice can inhibit the adhesion of 80% of uropathogenic *E. coli* on the urothelium for 10 h after ingestion [127]. A randomized placebo-controlled study in which older women were given 300 mL of cranberry juice containing 36 mg of proanthocyanidins or placebo showed a statistically significant reduction in the possibility of bacteriuria and pyuria of 42% in the group that received the cranberry juice [128]. However, since other studies have suggested that a higher amount of proanthocyanidins are needed for adequate and prolonged inhibition of bacterial adhesion, the optimal concentration and the best formulation remain unknown [22,129,130,131].

Several studies have evaluated the effect of cranberries in treating and preventing the recurrence of UTIs [131,132,133,134]. Importantly, a recent systematic review evaluated and critically appraised the clinical trials assessing the effect of cranberry use in the prevention and treatment of uncomplicated lower UTIs (cystitis) in healthy adult women [7]. In this systematic review, 12 such studies were included for further evaluation. Among them, eleven assessed the effect of different formulations of cranberries on the prevention of recurrent UTIs [135,136,137,138,139,140,141,142,143,144,145]. Ten of those were randomized, the mean or median age in all these studies was up to 55 years, and in most studies, the cranberry product was compared with placebo. In 6 of these 11 studies, the cranberry product was shown to reduce the likelihood of recurrent UTIs. At the same time, adverse events were rare and, in most cases, included gastrointestinal symptoms that, in a small number of women, led to discontinuation of the product [7]. Even though the success rate among these studies was generally inadequate, there were several issues regarding heterogeneity, differences in the proanthocyanidins concentration that was administered, and poor study design, thus limiting the generalizability of the results. Until now, cranberry products are not widely endorsed in guidelines for use in the prevention of UTIs but could be used on a case-by-case basis based on a conditional recommendation [114].

### 5.2. D-Mannose

**Figure 3:**
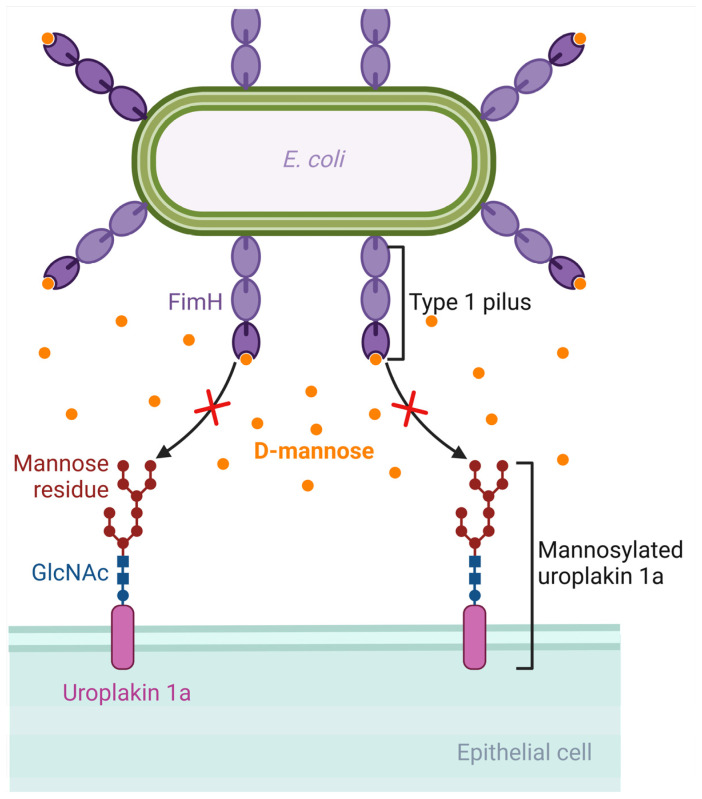
Mechanism of D-mannose inhibition of binding of Escherichia coli to the urothelium. D-mannose has a similar structure to the binding site of the glycoprotein receptors of the urothelium (such as uroplakin). If D-mannose is present in adequate concentrations in the urine, it can saturate FimH adhesin and inhibit bacterial binding to the urothelial receptors. Created with BioRender.com (license date: 28 May 2024).

D-mannose is a monosaccharide and an isomer of glucose. It is involved in the glycosylation of proteins such as monoclonal antibodies. Following oral administration, D-mannose is rapidly absorbed, can be detected in the plasma in about 30 min, and is excreted in the urine [146]. *E. coli* has virulence factors like pili, which are protein structures that extend from the bacterial cell and allow it to attach to host cells [147]. Type 1 pili, which are composed of Fim proteins, have an adhesion lectin domain that allows them to bind to mannosylated proteins of the host, such as uroplakin 1a, β1 and α3 integrins, and Tamm–Horsfall glycoproteins, which are located on the urothelium [22,148,149,150,151]. FimH, the protein that has the adhesion lectin domain responsible for binding to mannosylated proteins of the host, seems to be critical in the pathogenesis of UTIs, as confirmed in mouse models of UTIs by *E. coli*, implying that targeting this protein could be promising in designing potential therapies for UTIs [152]. Due to the similarity between D-mannose and the binding site of urothelial proteins such as uroplakins, an increased concentration of D-mannose in the urine could competitively inhibit the adhesion of the FimH protein on the mannosylated uroplakin receptors, thus preventing the binding of *E. coli* bacterial cells on these receptors [153]. For example, in a rat model of intravesical inoculation with *E. coli*, a 10% D-mannose solution was able to reduce bacteriuria compared to a control of normal saline [153]. Moreover, an in vitro study evaluating the ability of D-mannose to inhibit the adhesion of different *E. coli* strains isolated from urine, the vagina, and the perianal area of women with recurrent UTIs showed that D-mannose was able to inhibit the adhesion of more than 60% of these strains by at least 50%, with complete inhibition shown in the majority of them [154]. In that direction, there are efforts towards developing synthetic mannosides with a much higher affinity for the FimH ligand than D-mannose, such as M4284, which has a 100,000-fold higher binding affinity for FimH. In a mouse model of colonization by an *E. coli* strain isolated from humans with cystitis, oral administration of M4284 led to significantly reduced intestinal colonization, and M4284 was also able to treat UTI in mice when compared to vehicle control [155]. Thus, such synthetic mannosides could be tested in future studies at a preclinical and then at a clinical level to elucidate the role of FimH inhibitors as potential therapeutic agents for UTIs [22,156]. Figure 3 shows the mechanism by which D-mannose can inhibit the binding of *E. coli* on the urothelium.

Clinical evidence regarding the use of D-mannose in managing UTIs is much less than that regarding the use of cranberry products [157,158,159]. A recent systematic review evaluated and critically appraised the clinical trials assessing the effect of D-mannose use in managing uncomplicated lower UTI (cystitis) in healthy adult women [7]. Two randomized studies were eventually included in the analysis, the mean and median ages were within the range of 42 to 52 years, and the comparison included patients receiving D-mannose or antibiotics (either nitrofurantoin or the combination of trimethoprim and sulfamethoxazole) and, in one study, no treatment in a third group [160,161]. The treatment duration was six months in both studies, and a positive effect was observed in both studies. Adverse effects were uncommon and mainly included diarrhea in about 8% of patients. Importantly, even though in that systematic review the trials with D-mannose were relatively fewer than those with cranberries, the authors conclude that treatment with D-mannose or with a combination of D-mannose and antibiotics could be the most beneficial treatment for reducing recurrences of UTIs [7]. They do, however, conclude that randomized trials with blinding and significantly larger sample sizes are needed to confirm the results shown in their study [7]. Regarding the dose of D-mannose, as summarized in a recent Cochrane systematic review, different doses from 200 mg to 3 g have been used. However, different studies were not comparable due to the different dosing. In most studies, efficacious doses were those from 500 mg to 2 g (or 3 g with a de-escalation to 2 g) [162]. No clear recommendation exists until now regarding their use in recurrent UTIs [114].

## 6. Natural Compounds as Antibiotic-Sparing Agents in the Era of AMR

Despite antibiotics’ activity against infections such as UTIs, the possibility of pathogens developing AMR is a significant drawback. Notably, AMR may develop more frequently in patients having recently received antibiotics and may even develop during antibiotic treatment in some pathogens [163,164]. In particular, UTIs nowadays have increasing resistance [17,165]. Patients on antimicrobial prophylaxis for UTIs may present with breakthrough infections by bacteria with AMR [166]. Thus, using anti-infectives wisely in these patients may be a critical antimicrobial stewardship approach leading to reduced costs, fewer adverse events, and fewer breakthrough infections by resistant pathogens [167]. From that perspective, natural compounds such as cranberry products or D-mannose could be used as antibiotic-sparing agents, thus allowing clinicians to preserve antibiotics for more severe infections [168].

## 7. Conclusions

Anti-infective treatment of infectious diseases has been long considered the mainstay of treatment of infectious diseases. However, due to the development of AMR, non-anti-infective therapies have emerged as potential alternative options for treating infections. Natural products could be such an example, and they have been used for many years in managing recurrent UTIs. Cranberry products and D-mannose have been periodically evaluated and have provided, at times, some encouraging results, even though more studies with better randomized, controlled, and blind designs should be performed in the future to better establish their safety and efficacy in managing patients with recurrent UTIs. Until then, D-mannose and cranberry products could be used as antibiotic-sparing agents on a case-by-case basis in some patients suffering from recurrent UTIs.

## Figures and Tables

**Table 1:** Examples of studies providing data on the activity of flavonoids on common microorganisms.

Type of Flavonoid	Bacterial Species	Synergy with Antibiotic	Reference
Flavones	*Escherichia coli*, *Staphylococcus aureus*		[64]
Flavonols	*Escherichia coli*, *Staphylococcus aureus*		[64]
Isoflavones	*Escherichia coli*, *Staphylococcus aureus*		[64]
Apigenin (flavone)	*Bacillus subtilis*, *Micrococcus luteus*		[80]
Apigenin (flavone)	*Bacillus subtilis*, *Escherichia coli*, *Pseudomonas auruginosa*, *Proteus vulgaris*		[81]
Apigenin (flavone)	*Escherichia coli*, *Pseudomonas aeruginosa*, *Proteus mirabilis*, *Klebsiella pneumoniae*, *Salmonella typhimurium*, *Enterobacter aerogenes*, *Enterobacter cloacae*		[82,83]
Flavone	*Proteus vulgaris*, *Proteus mirabilis*		[84]
Flavone	Vancomycin-intermediate *Staphylococcus aureus*	Vancomycin, Oxacillin	[85]
Apigenin (flavone)	MRSA	Ampicillin, Ceftriaxone	[86]
Luteolin (flavone)	MRSA	Ampicillin, Cephradine, Ceftriaxone, Imipenem, Methicillin	[87]
Hesperetin (flavanon)	*Staphylococcus aureus*		[88]
Galangin (flavonol)	MRSA, MSSA *Enterococcus* spp., *Pseudomonas aeruginosa*		[89]
Luteolin (flavone)	*Streptococcus pyogenes*	Ceftazidime	[90]
Quercetin (flavonol)	*Staphylococcus aureus*	Cloxacillin	[91]
Quercetin (flavonol)	MRSA	Ceftriaxone	[87]
Quercetin (flavonol) + Luteolin (flavone)	MRSA Clinical Isolates	Imipenem	[87]
Genistein (isoflavone)	*Staphylococcus aureus*	Norfloxacin	[92]
EGCG—epigallocatechin gallate	MRSA6975, MRSA3202	Tetracycline Oxacillin	[93]

MRSA: methicillin-resistant *Staphylococcus aureus*; MSSA—methicillin-sensitive *Staphylococcus aureus*; the table may not be exhaustive of all studies in the literature.

## Data Availability

Not applicable.
